# Dietary branched-chain amino acids modulate the dynamics of calcium absorption and reabsorption in protein-restricted pigs

**DOI:** 10.1186/s40104-021-00669-w

**Published:** 2022-02-10

**Authors:** Mohammad Habibi, Cedrick N. Shili, Julia Sutton, Parniyan Goodarzi, Adel Pezeshki

**Affiliations:** Department of Animal and Food Sciences, Oklahoma State University, 206C Animal Science Building, Stillwater, OK 74078 USA

**Keywords:** Branched-chain amino acids, Calcium absorption and reabsorption, Calcium digestibility, Pig, Very low protein diets

## Abstract

**Background:**

Very low-protein (VLP) diets negatively impact calcium (Ca) metabolism and absorption. The objective of this study was to investigate the effect of supplemental branched-chain amino acids (BCAA) and limiting amino acids (LAA) on Ca digestibility, absorption and reabsorption in pigs fed with VLP diets. Forty-eight piglets were assigned to six treatments: positive control (PC), negative control (NC), and NC containing LAA 25%, LAA 50%, LAA + BCAA 25% (LB25) and LAA + BCAA 50% (LB50) more than recommendations.

**Results:**

Relative to PC or NC, LB25 and LB50 had higher digestibility of Ca and plasma Ca and phosphorus (P), but lower plasma vitamin D_3_. LB50 tended to increase vitamin D receptor transcript and protein in the gut, but decreased mRNA or protein abundance of parathyroid hormone 1 receptor (PTH1R), calbindin 1 (CALB1), cytochrome P450 family 27 subfamily B member 1 and occludin in small intestine. LB50 increased the transcript of cytochrome P450 family 24 subfamily A member 1 and *PTH1R* but decreased the transcript of transient receptor potential cation channel subfamily V member 5, *CALB1* and solute carrier family 17 member 4 in kidney.

**Conclusion:**

Overall, BCAA increased Ca digestibility through regulating the transcellular and paracellular Ca absorption in the gut and reabsorption in kidney during protein restriction.

## Background

The nitrogen (N) excretion from modern swine production adversely influences the environment and human health [1]. Very low protein (VLP) diets, i.e., diets with > 25% reduced crude protein (CP) produce beneficial outcome in terms of N excretion [2, 3]; however, these diets impair the growth of pigs despite the supplementation of limiting amino acids [LAA; i.e., lysine (Lys), methionine (Met), threonine (Thr), and tryptophan (Trp)] [2, 4–6]. This suggests that other amino acids (AA) may become limiting in these diets which requires further research to be identified when VLP diets are offered.

We recently showed that the branched-chain amino acid (BCAA) metabolism is significantly affected in pigs fed with VLP diets [4]. BCAA including leucine (Leu), isoleucine (Ile) and valine (Val) are essential AA, which are involved in energy homeostasis, muscle protein synthesis, immune function [7–9] and many other physiological processes . Supplementation of Val alone [10], Val and Ile [11] or all BCAA [7, 12–14] have been shown to improve the weight gain and feed efficiency in pigs fed with VLP diets. The BCAA-induced growth in pigs fed with VLP diets has been linked with improved muscle protein synthesis, blood amino acids profile, gut microbiota composition and feed intake [7, 11, 13–15]. Previous studies provide some evidence that the positive effects of BCAA on growth might be also related to their role on increasing the calcium (Ca) absorption or decreasing its excretion. In broilers, Val deficiency resulted in leg abnormalities, low bone Ca levels and increased Ca excretion in urine [16, 17]. In ovariectomized rats fed with low protein diets, a mixture of essential AA including BCAA improved the bone strength and bone mineral density [18]. Given the fact that low protein intake impairs the bone health [19, 20], reduces bone mineral density and bone mineral content [21] and digestibility of Ca [22, 23] and evidence on possible involvement of BCAA on Ca balance and absorption [16–18, 24], it is unknown whether supplementing VLP diets with BCAA would influence the dynamics of Ca (re)absorption. We recently showed that supplementation of BCAA higher than NRC recommendations partly annuls the negative effects of VLP diets on weight gain and feed intake [14]. To our knowledge, there are no previous reports on the effect of diets over-supplemented with BCAA on Ca digestibility during protein restriction.

Nutrients restriction [25] and protein deficiency [26] influences the expression of nutrients transporters in the gastrointestinal tract. Ca absorption has been reported to be improved in rats fed with diets supplemented with alanine (Ala), Ile, Leu, proline (Pro), and hydroxyproline [27]. BCAA have been shown to be involved in intestinal barrier function and nutrients absorption [28] and have stimulatory effects on digestive enzyme activity, expression of intestinal nutrients transporters, and abundance of beneficial gut bacteria in piglets [29]. However, the underlying mechanisms by which BCAA influence Ca digestibility, absorption, and metabolism have yet to be uncovered. Calcium homeostasis is tightly regulated with a complex system including circulatory hormones such as parathyroid hormone (PTH), calcitonin, 1,25-dihydroxyvitamin D, and fibroblast growth factor 23 (FGF-23), intestinal and renal transporters such as transient receptor potential cation channel subfamily V (TRPV), vitamin D receptor (VDR), solute carrier transporters, calbindin (CALB), and Ca-sensing receptors (CaSR) and gut paracellular transport proteins such as occludin (OCLN), zonula occludens 1 (ZO1), and claudins (CLDN) [30, 31]. It has been shown that apical Na^+^/H^+^ exchanger 3 (NHE3) inhibitor could suppress Leu-enhanced Ca transport and sodium efflux from basolateral membrane suggesting that the BCAA-enhanced Ca transport is dependent to NHE3 pathway [27]. The data on modulatory effect of BCAA on gut and kidney transporters and hormones associated with Ca and phosphorus (P) hemostasis and the significance of these interactions on BCAA induced growth are scarce. Therefore, the objective of this study was to investigate the effect of supplemental BCAA above recommended levels on Ca digestibility, hormones and metabolites and gene and protein expression of markers associated with Ca absorption and reabsorption in young pigs fed with VLP diets.

## Methods

### Animals, housing and diets

All procedures performed in the current study were in accordance with guidelines of Animal Care and Use Committee at Oklahoma State University and were approved by this committee (ACUP #AG-17-27). The animals, housing, diets and experimental design were previously described by authors [14]. Briefly, forty-eight three-week-old weaned barrows (Duroc sire line and Large White × Landrace dam; Seaboard, Hennessey, OK, USA) were individually housed in an environmentally controlled facility (26–30 °C) with 10-h light from 07:00 to 17:00 and 14-h half-light starting 17:00. Following two weeks of adaptation, pigs were weight-matched (9.0 ± 2.9 kg) and randomly assigned to six dietary treatments (8 pigs/treatment) for four weeks: 1) positive control (PC): basal diet with standard protein content [32]; 2) negative control (NC): basal diet with very low protein content containing LAA at NRC (2012) recommendations levels [32]; 3) NC containing LAA 25% more than NRC recommendation (L25); 4) NC containing LAA 50% more than NRC recommendation (L50); 5) NC containing both LAA and BCAA 25% more than NRC recommendation (LB25); 6) NC containing both LAA and BCAA 50% more than NRC recommendation (LB50) [14]. As recommended by NRC [32], pigs were fed with phase feeding approach to accurately meet the nutrient requirements. Three dietary phases were used including: nursery phase 1 (N1) for one week, nursery phase 2 (N2) for two weeks, and nursery phase 3 (N3) for three weeks. The authors previously reported [14] that the initial body weight was not different among dietary groups (PC 9.18 ± 0.49, NC 9.18 ± 0.49, L25 9.13 ± 0.49, L50 8.90 ± 0.49, LB25 9.01 ± 0.49, LB50 9.18 ± 0.49 kg; *P* = 0.99), but at the end of study the LB25 had higher body weight than L25 (14.26 ± 0.98 vs. 12.38 ± 0.98 kg, respectively). Further, LB50 had higher final body weight (15.62 ± 0.98 kg) than NC (13.22 ± 0.98 kg) and L50 (11.40 ± 0.91 kg) (*P* < 0.01). Chromium oxide (Alfa Aesar, Ward Hill, MA, USA) was supplemented to all diets as a marker for measuring the nutrients digestibility [14]. Experimental diets ingredients and composition have been given in our previous publication [14].

### Feed, fecal, blood, and tissue samples collection

After mixing the diets, feed samples from all bags of each treatment (~ 1 kg) were collected and stored at − 20 °C until analysis. Fecal grab samples were collected by rotational transfer of pigs from their individual pens to metabolic crates for 24 h during N2 and N3 phases. All pigs had free access to feed and water in metabolic crates. The collected samples were stored at − 20 °C until further analysis. Fecal consistency score was determined weekly with 0  =  formed, normal; 1  =  soft, wet cement consistency; 2  =  runny or watery; 3  =  mucoid diarrhea; 4  =  bloody diarrhea, as previously described [33]. As we described previously [14] at the end of the experiment, blood samples were taken from the anterior jugular vein of the pigs into lithium heparin and serum tubes when pigs were at the ad lib fed state (BD Vacutainer®, Franklin Lakes, NJ, USA). The collected blood samples were transported to the laboratory on ice and centrifuged at 3000 × *g* for 15 min at 4 °C. The collected plasma and serum samples were aliquoted and stored at − 80 °C until future analysis. Following blood collection, all pigs were euthanized by CO_2_ asphyxiation method, duodenum, jejunum, and kidney samples were collected, rinsed in distilled water, snap-frozen in liquid nitrogen and stored at − 80 °C until analysis.

### Chemical analysis of feed and feces, and apparent fecal digestibility of nutrients

As we described previously [14, 21], chemical analysis of the experimental diets for dry matter, CP, Ca, P and chromium was performed by Servi-Tech laboratories (Dodge City, KS, USA). Fecal samples were also analyzed for Ca, P, N and chromium content (Servi-Tech laboratories; Dodge City, KS, USA). Apparent fecal digestibility (AFD) of Ca, P and N was calculated using the following formula as we previously described [21]:

$$ AFD=100-\left[100\times \left( Crd/ Crf\right)\times \left( Nf/ Nd\right)\right]. $$

*Crd*: chromium amount in the diet, *Crf*: chromium amount in the feces, *Nf*: the nutrient amount in the feces, *Nd*: nutrient amount in the diet.

### Plasma calcium, phosphorus, alkaline phosphatase, and urea nitrogen

Concentration of Ca (Catalog no. BL251), P (Catalog no. BL218), alkaline phosphatase (ALP; Catalog no. BL206), and blood urea nitrogen (BUN; Catalog no. BL252) in plasma (> 300 μL) were detected using a chemistry analyzer system (CLC 480/BioLis24i, Carolina Liquid Chemistries Corp., Brea, CA, USA) following calibration (Catalog no. BL-442600, Multi-Analyte calibrator for Synchron CX/LX) [34]. The absorbance of Ca, P, ALP, and BUN were detected at 660, 340, 405, and 340 nm, respectively.

### Plasma vitamin D_3_ and fibroblast growth factor-23

Plasma vitamin D_3_ (VD_3_; Catalog no. PV0037, Porcine VD_3_ ELISA Kit, Kendall Scientific, IL, USA) and serum FGF-23 (Catalog no. EP0059, Porcine FGF-23 ELISA Kit, Wuhan Fine Biotech Co., Ltd., Wuhan, China) concentrations were measured according to manufacturers’ protocols. Using Epoch microplate spectrophotometer (BioTek® Instruments, Inc. Highland Park, VT, USA), the VD_3_ and FGF-23 absorbance were detected at 450 nm. The intra-assay coefficient of variation for VD_3_ and FGF-23 were 3.97% and 1.52%, respectively.

### RNA isolation, reverse transcription, and quantitative PCR

The relative mRNA abundances of key molecules involved in Ca and P absorption and excretion in duodenum and kidney were determined using quantitative PCR (qPCR) [35]. Briefly, total RNA was isolated from frozen tissues (~ 200 mg) by RNeasy® mini kit (Qiagen, Germantown, MD, USA) according to manufacturer’s instructions. The extracted RNA was quantified using a NanoDrop ND-1000 spectrophotometer (Thermo Fisher, Waltham, MA, USA). After treating the diluted RNA (250 ng/μL) with DNase I (Invitrogen, Carlsbad, CA, USA), the complementary DNA (cDNA) was synthesized using the following thermoscycler program (T100™ Thermal Cycler, Bio-Rad, Hercules, CA, USA): 22 °C for 5 min, 42 °C for 30 min, 85 °C for 5 min, and terminated at 4 °C. Next, the mRNA abundance of target genes was quantified using qPCR (CFX96 real time PCR detection system, Bio-Rad, Hercules, CA, USA) and primers for cytochrome P450 family 24 subfamily A member 1 (*CYP24A1*) [36], parathyroid hormone 1 receptor (*PTH1R*) [36], thyroid hormone receptor alpha (*THRA*) [36], solute carrier family 34 member 1 (*SLC34A1*) [36], solute carrier family 34 member 2 (*SLC34A2*) [36], solute carrier family 34 member 3 (*SLC34A3*) [36], solute carrier family 17 member 4 (*SLC17A4*) [37], transient receptor potential cation channel subfamily V member 5 (*TRPV5*) [38], transient receptor potential cation channel subfamily V member 6 (*TRPV6*) [39], *CALB1* [38], *VDR* [40], plasma membrane Ca^2+^ ATPase 1b (*PMCA1b*) [41], *OCLN* [42],* ZO1* [42], and β-Actin [43] (Table 1). The qPCR program used was as following: denaturation at 50 °C (2 min) and 95 °C (10 min), 40 cycles amplification: 95 °C (15 s) and 60 °C (1 min) followed by a melt curve program: 60 to 95 °C with the increment of 0.5 °C for 5 s. Finally, with Ct values for target and housekeeping (β-Actin) genes and using the 2^-∆∆CT^ method, the relative abundances of target genes transcript were obtained.

**Table 1:** The sequences [forward (F) and reverse (R)], location on template, amplicon size (bp), and GenBank accession numbers for primers used for reverse transcription quantitative real-time polymerase chain reaction (RT-qPCR)

Genes^**1**^	Sequence (5′ → 3′)	Location on template	Amplicon length, bp	GenBank accession no.
*CYP24A1*	**F:** GCTGGACAACAAAATCAATGAG	898–919	142	NM_214075.2
	**R:** CTCATAGAGCACAAGGCAGATG	1018–1039		
*PTH1R*	**F:** GGCGTCCATTACATCGTCTT	1390–1409	198	NM_214382.1
	**R:** AAAGTCCAGTGCCAATGTCC	1568–1587		
*THRA*	**F:** TGGAAAGCGAAAAAGAAAGAAC	205–226	202	NM_214190.1
	**R:** TGAGTAGGTGGGATGGAGATTC	385–406		
*SLC34A1*	**F:** TGGGCTTGTGTGACTGAGAG	2124–2143	198	NM_001044623.1
	**R:** CCCAGTCAGAGTTGTGCGTA	2302–2321		
*SLC34A2*	**F:** GAATCAGCCCGAAACAAGAG	835–854	128	NM_001256772.1
	**R:** AAACCATCCGTCCAACAGAG	943–962		
*SLC34A3*	**F:** TCGTCCTGGTCACAGTCC	2204–2221	192	XM_021081180.1
	**R:** CGGGGTTCTCATAGCAGTG	2377–2395		
*SLC17A4*	**F:** TTTTCAATTTCCACCCAACAAAT	1834–1856	76	XM_021098408.1
	**R:** GGGTGGGCAGAGCTGTGT	1892–1906		
*TRPV5*	**F:** AGGGTCGGTTTCTCTCGCTA	1437–1456	75	XM_021078896.1
	**R:** GGCATAGGTGATGGTGATGACA	1490–1511		
*TRPV6*	**F:** CACTTTAGGAGAGGCTTGCTG	2845–2865	147	XM_003134594.1
	**R:** ATGACTTTATTGGAAGGTAGGAGGT	2967–2991		
*CALB1*	**F:** ACGCTGACGGAAGTGGTTAC	77–96	84	NM_001130226.1
	**R:** ATCCAGCCTTCTTTCGTGCC	141–160		
*VDR*	**F:** AGGCTTCTTCAGACGGAGCATGAA	285–308	140	XM_021091108.1
	**R:** ACTCCTTCATGCCGATGTCCA	404–424		
*PMCA1b*	**F:** TCTAAAAGAAGCTGGTCATGGAACAC	3684–3709	363	XM_021091182.1
	**R:** TCTTGATTCTGGCTTTTCTAACCCT	4022–4046		
*OCLN*	**F:** TCCTGGGTGTGATGGTGTTC	633–652	144	NM_001163647.2
	**R:** CGTAGAGTCCAGTCACCGCA	757–776		
*ZO1*	**F:** AAGCCCTAAGTTCAATCACAATCT	4981–5004	130	XM_021098827.1
	**R:** ATCAAACTCAGGAGGCGGC	5092–5110		
β-Actin	**F:** CTGCGGCATCCACGAAACT	944–962	147	XM_021086047.1
	**R:** AGGGCCGTGATCTCCTTCTG	1071–1090		

^1^ *CYP24A1*, cytochrome P450 family 24 subfamily A member 1;* PTH1R*, parathyroid hormone 1 receptor; *THRA*, thyroid hormone receptor alpha; *SLC34A1*, solute carrier family 34 member 1; *SLC34A2*, solute carrier family 34 member 2;* SLC34A3*, solute carrier family 34 member 3; *SLC17A4*, solute carrier family 17 member 4; *TRPV5*, transient receptor potential cation channel subfamily V member 5; *TRPV6*, transient receptor potential cation channel subfamily V member 6; *CALB1*, calbindin-1; *VDR*, vitamin D receptor; *PMCA1b*, plasma membrane Ca ATPase 1b; *OCLN*, occludin; *ZO1*, zonula occludens 1

### Immunoblot analysis

Western blot was performed for NHE3, CLDN-1, VDR, and cytochrome P450 family 27 subfamily B member 1 (CYP27B1) in jejunum and kidney [44, 45]. Briefly, jejunum and kidney samples (300–500 mg) were ground in liquid nitrogen and homogenized in the mixture of NP40 buffer (Life Technologies, MD, USA), protease inhibitor cocktail (Bio-World, OH, USA) and phenylmethylsulfonyl fluoride (Acros Organics, NJ, USA). Following homogenization, brief sonication, and centrifugation (2500 × *g* for 15 min), protein concentration of samples was determined using Bradford assay. Then, protein homogenates were mixed with 2 × Laemmli buffer (Sigma-Aldrich, MO, USA) and incubated at 95 °C for 3 min. The protein extract (40 μg; 2 mg/mL) derived from each sample was fractioned using 10% sodium dodecyl sulfate polyacrylamide gel electrophoresis (Fisher Scientific, NJ, USA). The constant voltage of 130 V was applied for gel electrophoresis for 1:15 h. After separation, the proteins were transferred into the nitrocellulose membranes applying the voltage of 100 V for 1:30 h. The membranes were blocked with 8% (m/v) skim milk in Tris-buffered saline containing 0.1% Tween-20 (TBST) for 2 h at room temperature with mild agitation. Then, the membranes were incubated with added primary antibodies (Table 2) in 8% skim milk (m/v) in TBST at 4 °C overnight. On the next day, the blots were washed for 20 min (3 ×) in TBST and incubated with secondary antibody (Table 3) in TBST-8% milk (1 h at room temperature). Then, blots were washed in TBST for 15 min (4 ×). Finally, protein bands were developed using Lumi-Light Western Blotting Substrate (Novex® ECL HRP Chemiluminescent Substrate Reagent Kit, Invitrogen, CA, USA) and captured using a ChemiDoc XR imaging system (Bio-Rad Laboratories Inc., CA, USA). The bands were quantified using Image Lab software (Version 6.0.1, Bio-Rad Laboratories Inc., CA, USA). To determine the relative quantity of target proteins, the GAPDH was used as a loading control.

**Table 2:** The host, dilution, and supplier of primary and secondary antibodies for immunoblotting

Antibodies	Host	Dilution	Vendor
Anti-NHE3^1^	Rabbit	1:375	ThermoFisher Scientific, Rockford, IL, USA #27190–1-AP
Anti-VDR^1^	Rat	1:150	ThermoFisher Scientific, Rockford, IL, USA #MA5–14614
Anti-CLDN-1	Rabbit	1:300	ThermoFisher Scientific, Rockford, IL, USA #71–7800
Anti-CYP27B1^1^	Rabbit	1:2000	ThermoFisher Scientific, Rockford, IL, USA #PA5–79128
Anti-GAPDH	Mouse	1:7500	Abcam, Cambridge, MA, USA #ab105428
Anti-Rabbit IgG H&L (HRP)	Goat	1:7500	Abcam, Cambridge, MA, USA #ab205718
Anti-Rat IgG (HRP)	Rabbit	1:3000	Abcam, Cambridge, MA, USA #ab6734

^1^ *NHE3*, Na^+^/H^+^ exchanger 3; *VDR*, vitamin D receptor; *CLDN-1*, claudin-1; *CYP27B1*, cytochrome P450 family 27 subfamily B member 1

**Table 3:** Effect of very low protein diets supplemented with limiting and branched-chain amino acids higher than recommended levels on fecal consistency score of nursery pigs

Weeks	Diets^**1**^						SEM^**2**^	***P***-value
	PC	NC	L25	L50	LB25	LB50		
Week 1	1.50 ± 0.20	1.37 ± 0.20	1.37 ± 0.20	1.37 ± 0.20	1.00^a$*^ ± 0.22	0.50^b¥^ ± 0.20	0.09	0.012
Week 2	1.00 ± 0.23	0.37 ± 0.23	0.50 ± 0.23	0.50 ± 0.23	0.75 ± 0.23	0.50 ± 0.23	0.09	0.420
Week 3	0.25 ± 0.16	0.12 ± 0.16	0.37 ± 0.16	0.29 ± 0.17	0.37 ± 0.16	0.12 ± 0.16	0.06	0.779
Week 4	0.29 ± 0.13	0.25 ± 0.12	0.50 ± 0.12	0.0 ± 0.13	0.0 ± 0.12	0.0 ± 0.12	0.06	0.033

^1^ *PC* (positive control), standard protein diet; *NC* (negative control), low protein diet supplemented with limiting amino acids (*LAA*, i.e., Lys, Met, Thr and Trp); *L25*, low protein diet supplemented with LAA, 25% more than NRC (2012) requirements; *L50*: low protein diet supplemented with LAA 50% more than NRC requirements; *LB25*: low protein diets supplemented with LAA and branched-chain amino acids (BCAA, i.e., Leu, Ile and Val) 25% more than NRC requirements; *LB50*: low protein diet supplemented with LAA and BCAA 50% more than NRC requirements. Fecal samples were scored according to: 0 = formed, normal; 1 = soft, wet cement consistency; 2 = runny or watery; 3 = mucoid diarrhea; 4 = bloody diarrhea. The *P-*values for the overall model effect for diet, week and diet × week were 0.053, < 0.01 and 0.13 respectively. ^a^ *P* ≤ 0.05 LB25 vs. PC, ^b^ *P* ≤ 0.05 LB50 vs. PC, ^$^ *P* ≤ 0.1 LB25 vs. NC, ^*^ *P* ≤ 0.1 LB25 vs. L25, ^¥^ *P* ≤ 0.1 LB50 vs. L50. The values are the means ± SE. *n* = 8

^2^* SEM*: standard errors of means

### Statistical analysis

As we previously described [4, 14], for analysis of nutrient digestibility, fecal consistency score, and all other data collected from laboratory analyses including plasma metabolites, qPCR and western blot data, outlier test followed by the GLM procedure (IBM SPSS Statistics Version 23, Armonk, NY, USA) was performed. For weekly fecal score and nutrient digestibility data in N2 and N3 phases, the mixed procedure was applied with the diet, time and the interaction of diet by time as fixed effects and the animal as a random variable. Based on the lowest levels of fit statistics for corrected Akaike Information Criterion and Bayesian Information Criterion, the modeling of covariance structure for the repeated measurements for each variable was performed. Differences among treatment means were separated using paired Student’s *t-*test followed by a Benjamini-Hochberg correction with 0.1 false discovery rate for six preplanned comparisons including PC vs. LB25, PC vs. LB50, NC vs. LB25, NC vs. LB50, L25 vs. LB25, and L50 vs. LB50. All values were presented as mean ± standard error of the mean with a *P* ≤ 0.05 considered statistically significant and 0.05 < *P* ≤ 0.1 considered as tendency.

## Results

### Apparent total tract digestibility of calcium, phosphorus and nitrogen

**Fig. 1:**
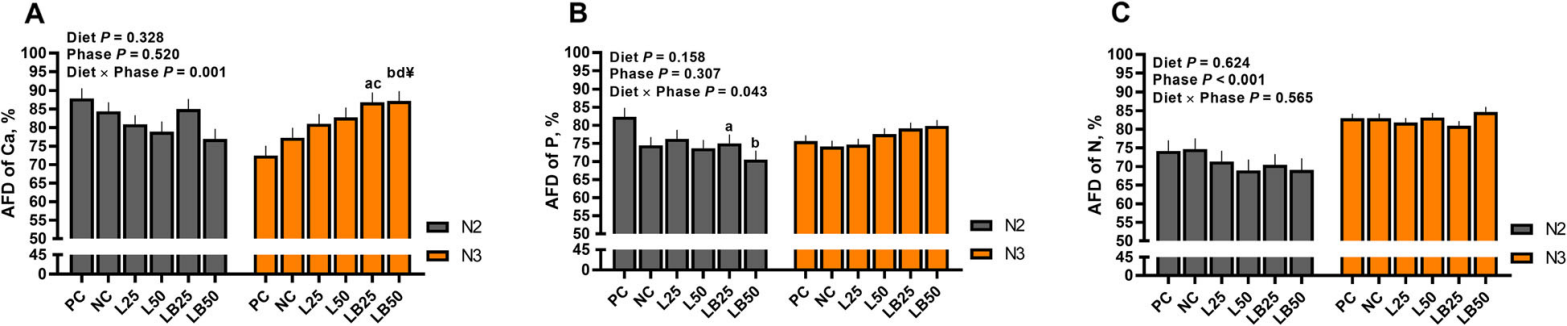
Effect of very low protein diets supplemented with limiting and branched-chain amino acids higher than recommended levels on apparent fecal digestibility of calcium, phosphorus, and nitrogen in nursery pigs. (**A**) apparent fecal digestibility (AFD) of calcium (Ca), (**B**) AFD of phosphorus (P), and (**C**) AFD of nitrogen (N) during nursery phase 2 (N2, i.e., days 15–21 of study) and nursery phase 3 (N3, i.e., days 22–42 of study) periods. PC (positive control), standard protein diet; NC (negative control), low protein diet supplemented with limiting amino acids (LAA, i.e., Lys, Met, Thr and Trp); L25, low protein diet supplemented with LAA, 25% more than NRC (2012) requirements; L50: low protein diet supplemented with LAA 50% more than NRC requirements; LB25: low protein diets supplemented with LAA and branched-chain amino acids (BCAA, i.e., Leu, Ile and Val) 25% more than NRC requirements; LB50: low protein diet supplemented with LAA and BCAA 50% more than NRC requirements. The *P*-values for the diet effects for N2 and N3 phases for AFD of Ca were 0.071 and 0.003, for AFD of P were 0.055 and 0.088 and for AFD of N were 0.619 and 0.457, respectively. ^a^ *P* ≤ 0.05 LB25 vs. PC, ^b^ *P* ≤ 0.05 LB50 vs. PC, ^c^ *P* ≤ 0.05 LB25 vs. NC, ^d^ *P* ≤ 0.05 LB50 vs. NC. ^¥^ 0.05 < *P* ≤ 0.1 LB50 vs. L50. The values are means ± standard error of the mean, *n* = 6

The effect of interaction of diet by phase on AFD of Ca was significant (Fig. 1A). The effect of the diet on AFD of Ca during N2 tended to be significant (*P* = 0.07), whereas dietary treatments significantly affected AFD of Ca in N3 phase (*P* ≤ 0.01; Fig. 1A). The mean values for AFD of Ca during N2 phase for PC, NC, L25, L50, LB25, and LB50 were 87.9%, 84.4%, 80.9%, 78.92%, 85.02%, and 76.9%, respectively. Compared to PC, AFD of Ca was 20% higher in pigs fed with LB25 and LB50 in N3 phase. Furthermore, LB25 and LB50 had higher (~ 13%) AFD of Ca than NC and LB50 tended to have higher AFD of Ca (~ 5%) relative to L50 during N3 phase (Fig. 1A).

Similar to AFD of Ca, the effect of interaction of diet by phase on AFD of P was significant (Fig. 1B). The effect of diet on AFD of P in N2 was significant (*P* = 0.05) and that tended to be significant in N3 (*P* = 0.09). While AFD of P was ~ 9% and 14% lower in LB25 and LB50 than PC during N2 (Fig. 1B), that was higher for these groups compared to the rest treatments during N3 (the mean values for AFD of P during N3 were 75.6%, 74.1%, 74.7%, 77.6%, 79.1% and 79.8% for PC, NC, L25, L50, LB25, and LB50, respectively; Fig. 1B).

The effects of diet and the interaction of diet by phase on AFD of N were not significant; however, the effect of phase was significant (Fig. 1C). The AFD of N were 71.4% and 82.8% during N2 and N3 phases, respectively. No differences among diets on AFD of N were detected within N2 and N3 phases (Fig. 1C).

### Fecal consistency

The overall effect of diet on fecal consistency score was significant. The mean fecal consistency scores were 0.77, 0.53, 0.69, 0.57, 0.50, and 0.28 for PC, NC, L25, L50, LB25, and LB50, respectively (Table 3). On week 1, fecal score was 34% and 67% lower in LB25 and LB50 than PC and it tended to be lower in LB25 (27%) than NC and L25 (Table 3). Furthermore, pigs fed with LB50 tended to have a 63% lower fecal score in comparison with L50 on week 1 (Table 3). No significant differences on fecal consistency score were detected across groups on week 2 and 3. Although the effect of diet on fecal consistency score was significant on week 4, no significant difference was detected among diets (Table 3).

### Plasma alkaline phosphatase, urea nitrogen, calcium, and phosphorus

**Fig. 2:**
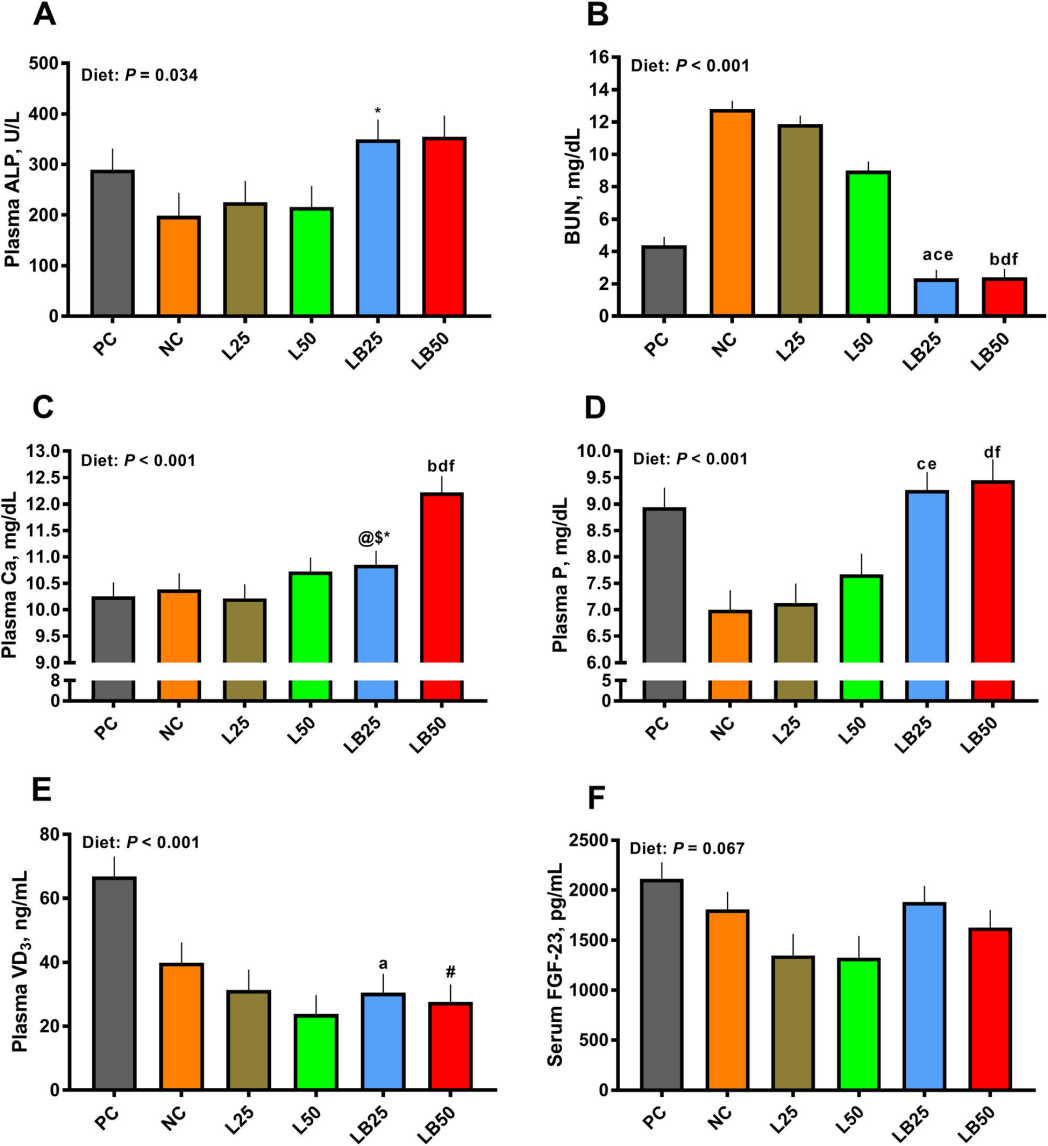
Effect of very low protein diets supplemented with limiting and branched-chain amino acids higher than recommended levels on blood metabolites and hormones. (**A**) plasma alkaline phosphatase (ALP), (**B**) blood urea nitrogen (BUN), (**C**) plasma calcium (Ca), (**D**) plasma phosphorus (P), (**E**) plasma vitamin D_3_ (VD_3_) and (**F**) serum fibroblast growth factor-23 (FGF-23). PC (positive control), standard protein diet; NC (negative control), low protein diet supplemented with limiting amino acids (LAA, i.e., Lys, Met, Thr and Trp); L25, low protein diet supplemented with LAA, 25% more than NRC (2012) requirements; L50: low protein diet supplemented with LAA 50% more than NRC requirements; LB25: low protein diets supplemented with LAA and branched-chain amino acids (BCAA, i.e., Leu, Ile and Val) 25% more than NRC requirements; LB50: low protein diet supplemented with LAA and BCAA 50% more than NRC requirements. ^a^ *P* ≤ 0.05 LB25 vs. PC, ^b^ *P* ≤ 0.05 LB50 vs. PC, ^c^ *P* ≤ 0.05 LB25 vs. NC, ^d^ *P* ≤ 0.05 LB50 vs. NC, ^e^ *P* ≤ 0.05 LB25 vs. L25, ^f^ *P* ≤ 0.05 LB50 vs. L50. ^@^ 0.05 < *P* ≤ 0.1 LB25 vs. PC, ^#^ 0.05 < *P* ≤ 0.1 LB50 vs. PC, ^$^ 0.05 < *P* ≤ 0.1 LB25 vs. NC, ^*^ 0.05 < *P* ≤ 0.1 LB25 vs. L25. The values are means ± standard error of the mean, *n* = 8

Overall, the effect of diet on plasma ALP was significant (*P* = 0.03; Fig. 2A). Plasma ALP tended to be higher by 55% in LB25 relative to L25 (Fig. 2A). Compared to PC, LB25 and LB50 had 46% and 44% lower BUN, respectively (Fig. 2B). LB25 and LB50 had 82% and 81% lower BUN relative to NC, respectively (Fig. 2B). Moreover, LB25 and LB50 pigs had 80% and 73% lower BUN than L25 and L50 pigs, respectively (Fig. 2B). Relative to PC, NC, and L25, plasma Ca concentration tended to be higher by 6%, 4%, and 6% in LB25, respectively (Fig. 2C). Moreover, pigs fed with LB50 had 19%, 17%, and 14% higher plasma Ca concentration than PC, NC, and L50, respectively (Fig. 2C). Compared to NC, LB25 and LB50 increased plasma P by 32% and 35%, respectively (Fig. 2D). Moreover, LB25 and LB50 increased plasma P by 30% and 23% relative to L25 and L50, respectively (Fig. 2D).

### Plasma VD_3_ and serum FGF-23

In comparison with PC, plasma VD_3_ was 54% and 59% lower in LB25 and LB50, respectively (Fig. 2E). The effect of diet on serum FGF-23 tended to be significant with 2114, 1806, 1348, 1327, 1879, and 1625 pg/mL for PC, NC, L25, L50, LB25, and LB50, respectively (Fig. 2F). No significant differences on serum FGF-23 concentration were observed among dietary treatments.

### The mRNA abundance of key molecules involved in Ca absorption in duodenum

**Fig. 3:**
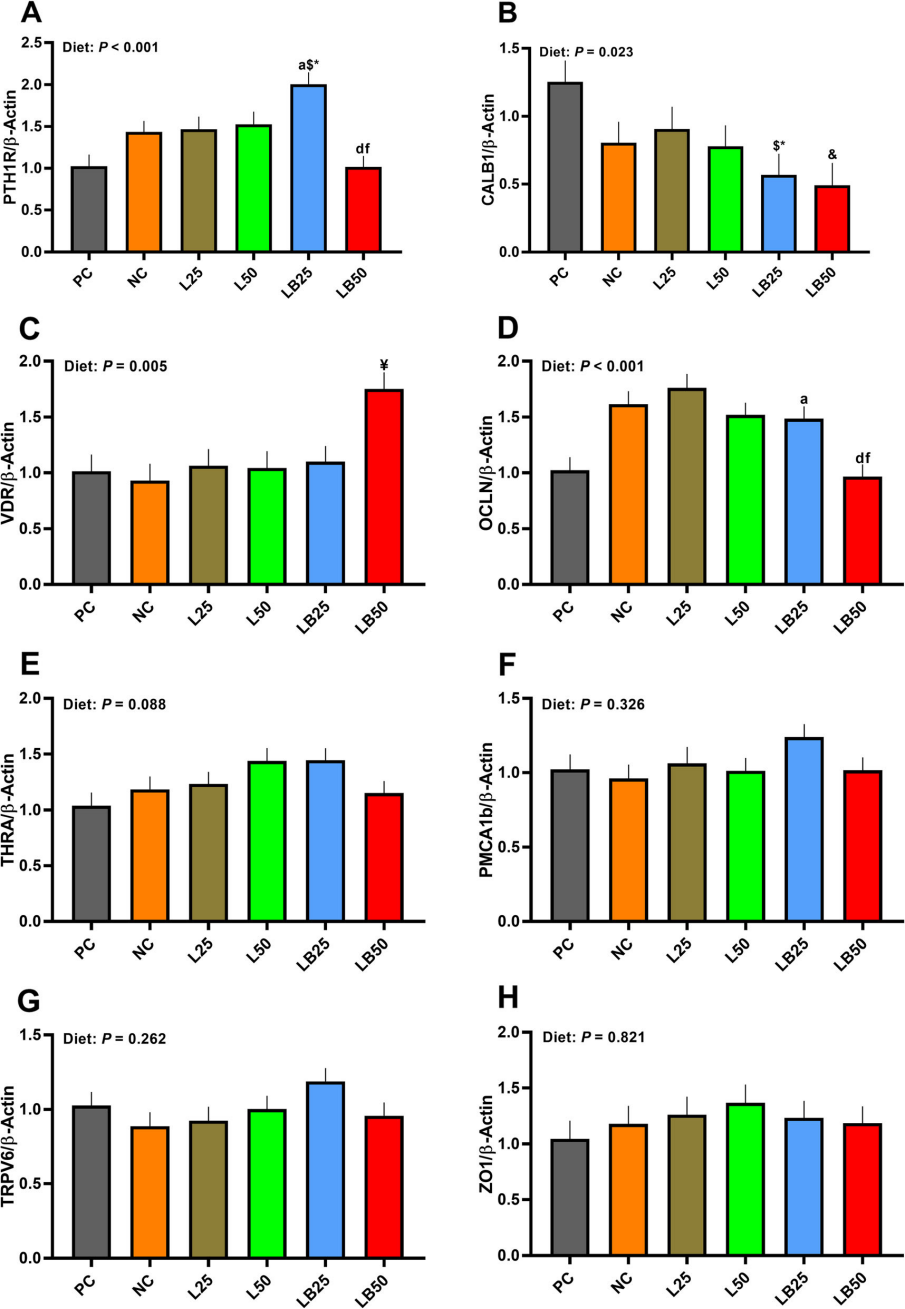
Effect of very low protein diets supplemented with limiting and branched-chain amino acids higher than recommended levels on mRNA abundance of markers associated with Ca/P absorption in duodenum. (**A**) parathyroid hormone 1 receptor (*PTH1R*), (**B**) calbindin 1 (*CALB1*), (**C**) vitamin D receptor (*VDR*) (**D**) occludin (*OCLN*), (**E**) thyroid hormone receptor alpha (*THRA*), (**F**) plasma membrane Ca^2+^ ATPase 1b (*PMCA1b*), (**G**) transient receptor potential cation channel subfamily V member 6 (*TRPV6*), (**H**) zonula occludens 1 (*ZO1*). PC (positive control), standard protein diet; NC (negative control), low protein diet supplemented with limiting amino acids (LAA, i.e., Lys, Met, Thr and Trp); L25, low protein diet supplemented with LAA, 25% more than NRC (2012) requirements; L50: low protein diet supplemented with LAA 50% more than NRC requirements; LB25: low protein diets supplemented with LAA and branched-chain amino acids (BCAA, i.e., Leu, Ile and Val) 25% more than NRC requirements; LB50: low protein diet supplemented with LAA and BCAA 50% more than NRC requirements. ^a^ *P* ≤ 0.05 LB25 vs. PC, ^d^ *P* ≤ 0.05 LB50 vs. NC, ^f^ *P* ≤ 0.05 LB50 vs. L50. ^$^ 0.05 < *P* ≤ 0.1 LB25 vs. NC, ^&^ 0.05 < *P* ≤ 0.1 LB50 vs. NC, ^*^ 0.05 < *P* ≤ 0.1 LB25 vs. L25, ^¥^ 0.05 < *P* ≤ 0.1 LB50 vs. L50. The values are means ± standard error of the mean, *n* = 8

The mRNA abundance of *PTH1R* was 95% higher in LB25 than PC and tended to increase by 40% and 37% in LB25 compared with NC and L25, respectively. However, the *PTH1R* transcript was 29% and 33% lower in LB50 than NC and L50, respectively (Fig. 3A). The transcript of *CALB1* tended to be lower in LB25 compared to L25 and NC (37% and 29%, respectively; Fig. 3B). LB50 also tended to have a 39% lower mRNA abundance of *CALB1* relative to NC. The mRNA abundance of *VDR* tended to increase by 68% in LB50 in comparison with L50 (Fig. 3C). The mRNA expression of *OCLN* was increased by 45% in LB25 compared to PC. Further, *OCLN* transcript was reduced in LB50 relative to NC and L50 (40% and 36%, respectively; Fig. 3D). The effect of diet on transcript of *THRA* tended to be significant, but with no significant differences across groups (Fig. 3E). No differences among treatments were detected for mRNA expression of *PMCA1b*, *TRPV6*, and *ZO1* (Fig. 3F–H).

### The mRNA abundance of key molecules involved in Ca/P reabsorption in kidney

**Fig. 4:**
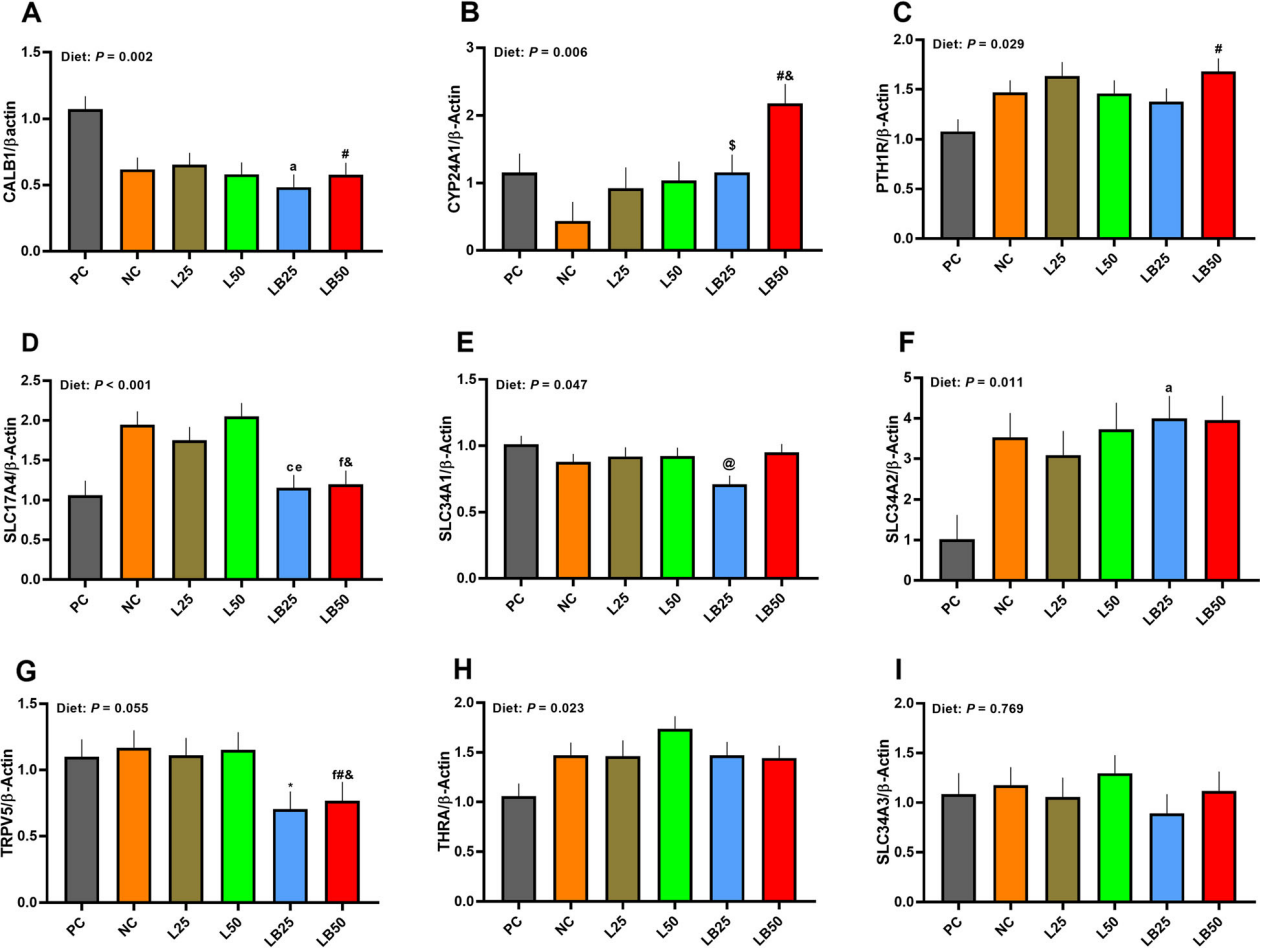
Effect of very low protein diets supplemented with limiting and branched-chain amino acids higher than recommended levels on mRNA abundance of markers associated with Ca/P reabsorption in kidney. (**A**) calbindin 1 (*CALB1*), (**B**) cytochrome P450 family 24 subfamily A member 1 (*CYP24A1*), (**C**) parathyroid hormone 1 receptor (*PTH1R*), (**D**) solute carrier family 17 member 4 (*SLC17A4*), (**E**) solute carrier family 34 member 1 (*SLC34A1*), (**F**) solute carrier family 34 member 2 (*SLC34A2*), (**G**) transient receptor potential cation channel subfamily V member 5 (*TRPV5*), (**H**) thyroid hormone receptor alpha (*THRA*), (**I**) solute carrier family 34 member 3 (*SLC34A3*). PC (positive control), standard protein diet; NC (negative control), low protein diet supplemented with limiting amino acids (LAA, i.e., Lys, Met, Thr and Trp); L25, low protein diet supplemented with LAA, 25% more than NRC (2012) requirements; L50: low protein diet supplemented with LAA 50% more than NRC requirements; LB25: low protein diets supplemented with LAA and branched-chain amino acids (BCAA, i.e., Leu, Ile and Val) 25% more than NRC requirements; LB50: low protein diet supplemented with LAA and BCAA 50% more than NRC requirements. ^a^ *P* ≤ 0.05 LB25 vs. PC, ^c^ *P* ≤ 0.05 LB25 vs. NC, ^e^ *P* ≤ 0.05 LB25 vs. L25, ^f^ *P* ≤ 0.05 LB50 vs. L50. ^@^ 0.05 < *P* ≤ 0.1 LB25 vs. PC, ^#^ 0.05 < *P* ≤ 0.1 LB50 vs. PC, ^$^ 0.05 < *P* ≤ 0.1 LB25 vs. NC, ^&^ 0.05 < *P* ≤ 0.1 LB50 vs. NC, ^*^ 0.05 < *P* ≤ 0.1 LB25 vs. L25. The values are means ± standard error of the mean, *n* = 8

The mRNA expression of renal *CALB1* was lower in LB25 and tended to be lower in LB50 than PC (55% and 46%, respectively; Fig. 4A). The mRNA abundance of *CYP24A1* tended to be higher in LB25 and LB50 than NC (165% and 398%, respectively); and it tended to be higher in LB50 relative to PC (89%; Fig. 4B). The transcript of renal *PTH1R* tended to be higher in LB50 than PC (56%; Fig. 4C). The mRNA abundance of *SLC17A4* was 34% and 41% lower in LB25 and LB50 than L25 and L50, respectively, and compared to NC, it was lower in LB25 and tended to be lower in LB50 (41% and 38%, respectively; Fig. 4D). While the mRNA abundance of renal *SLC34A1* tended to be 30% lower in LB25 than PC (Fig. 4E), the renal *SLC34A2* mRNA abundance was 291% higher in LB25 compared to PC (Fig. 4F). The transcript of renal *TRPV5* was 33% lower in LB50 compared to L50 and it tended to be 30% and 34% lower in LB50 relative to PC and NC, respectively (Fig. 4G). Furthermore, renal *TRPV5* transcript tended to be 36% lower in LB25 than L25 (Fig. 4G). Although the overall effect of the diet on mRNA abundance of renal *THRA* was significant, it did not change across the diets (Fig. 4H). No differences among diets were detected for the mRNA abundance of renal *SLC34A3* (Fig. 4I).

### The protein abundance of key molecules involved in Ca absorption in jejunum

**Fig. 5:**
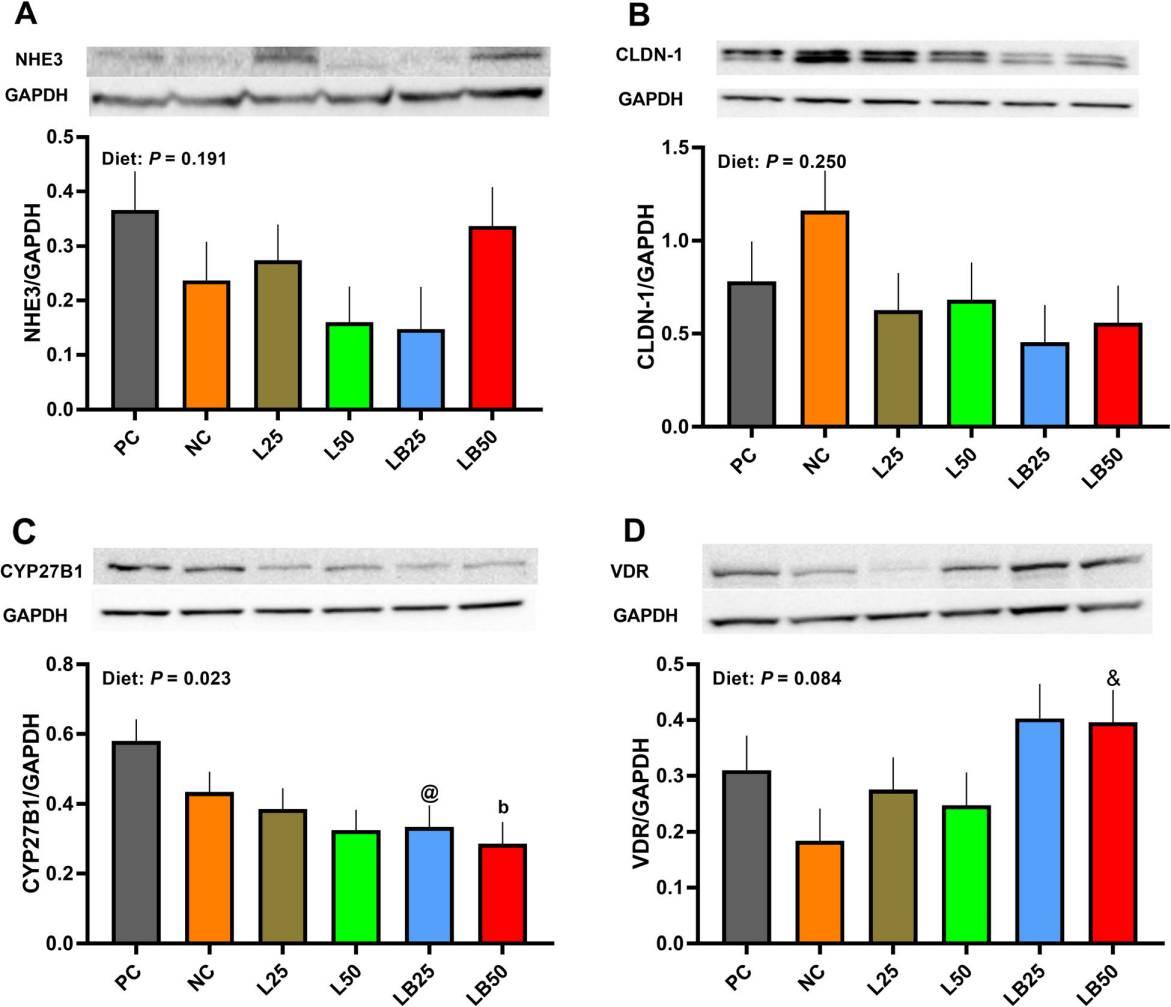
Effect of very low protein diets supplemented with limiting and branched-chain amino acids higher than recommended levels on protein abundance of markers associated with Ca/P absorption in jejunum. (**A**) Na^+^/H^+^ exchanger 3 (NHE3), (**B**) claudin-1 (CLDN-1), (**C**) cytochrome P450 family 27 subfamily B member 1 (CYP27B1), (**D**) vitamin D receptor (VDR). PC (positive control), standard protein diet; NC (negative control), low protein diet supplemented with limiting amino acids (LAA, i.e., Lys, Met, Thr and Trp); L25, low protein diet supplemented with LAA, 25% more than NRC (2012) requirements; L50: low protein diet supplemented with LAA 50% more than NRC requirements; LB25: low protein diets supplemented with LAA and branched-chain amino acids (BCAA, i.e., Leu, Ile and Val) 25% more than NRC requirements; LB50: low protein diet supplemented with LAA and BCAA 50% more than NRC requirements. ^b^ *P* ≤ 0.05 LB50 vs. PC, ^@^ 0.05 < *P* ≤ 0.1 LB25 vs. PC, ^&^ 0.05 < *P* ≤ 0.1 LB50 vs. NC. The values are means ± standard error of the mean, *n* = 8

Compared to PC, the protein abundance of jejunal CYP27B1 was 50.7% lower in LB50, and it tended to be lower (42.4%) in LB25 (Fig. 5C). Jejunal VDR protein abundance tended to increase by 115% in LB50 relative to NC (Fig. 5D). The protein abundance of jejunal NHE3 and CLDN-1 was not different among dietary treatments (Fig. 5A–B).

### The protein abundance of key molecules involved in Ca/P reabsorption in kidney

**Fig. 6:**
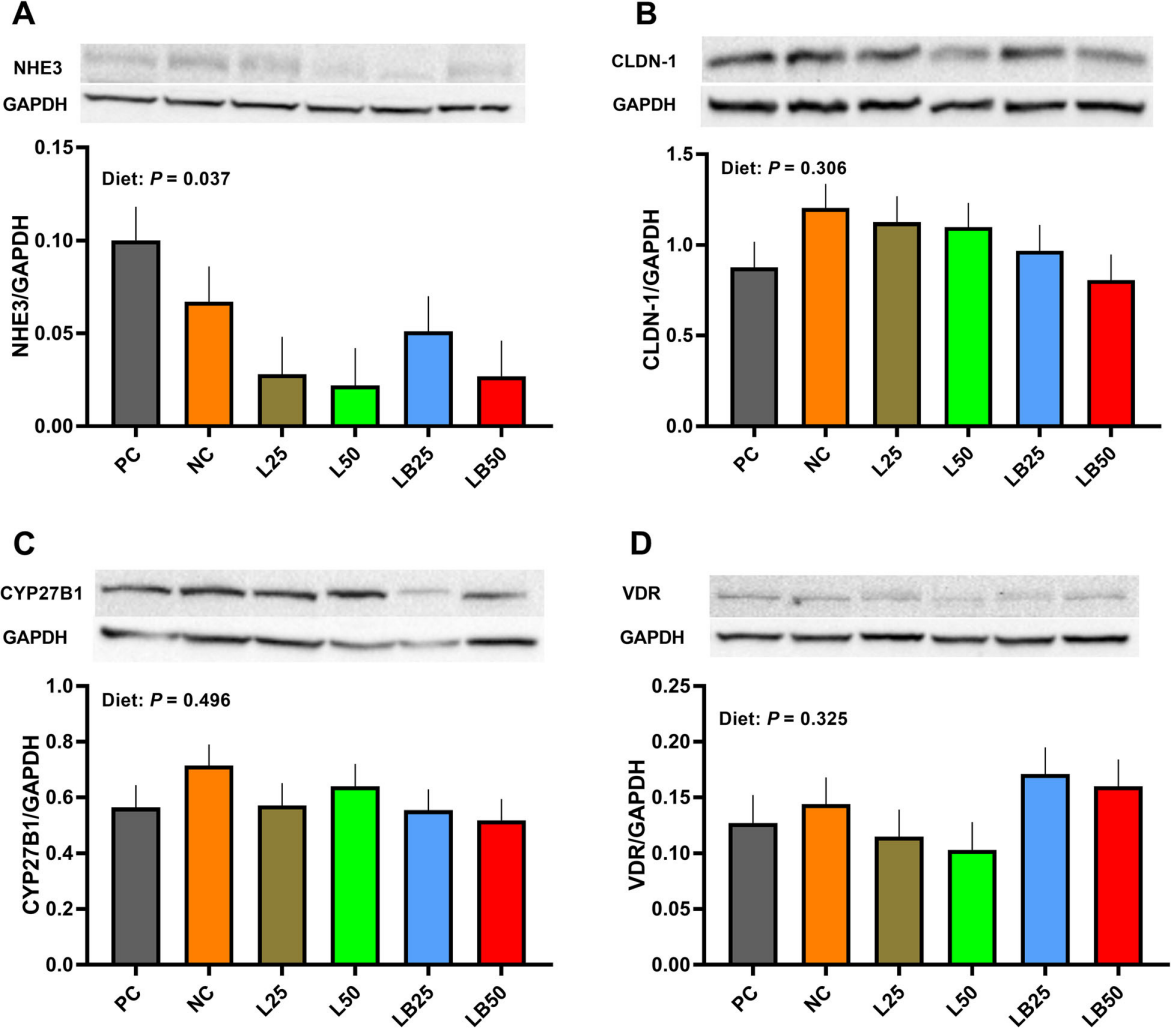
Effect of very low protein diets supplemented with limiting and branched-chain amino acids higher than recommended levels on protein abundance of markers associated with Ca/P reabsorption in kidney. (**A**) Na^+^/H^+^ exchanger 3 (NHE3), (**B**) claudin-1 (CLDN-1), (**C**) cytochrome P450 family 27 subfamily B member 1 (CYP27B1), (**D**) vitamin D receptor (VDR). PC (positive control), standard protein diet; NC (negative control), low protein diet supplemented with limiting amino acids (LAA, i.e., Lys, Met, Thr and Trp); L25, low protein diet supplemented with LAA, 25% more than NRC (2012) requirements; L50: low protein diet supplemented with LAA 50% more than NRC requirements; LB25: low protein diets supplemented with LAA and branched-chain amino acids (BCAA, i.e., Leu, Ile and Val) 25% more than NRC requirements; LB50: low protein diet supplemented with LAA and BCAA 50% more than NRC requirements. The values are means ± standard error of the mean, *n* = 8

The effect of diet on relative protein abundance of renal NHE3 was significant; however, no significant differences were found among diets (Fig. 6A). The protein abundance of renal CLDN-1, CYP27B1, and VDR did not change among dietary treatments (Fig. 6B–D).

## Discussion

Feeding pigs with VLP diets is an effective dietary strategy to reduce N excretion from modern swine production [2, 3]; however, these diets impair their growth despite the supplementation of LAA [2, 4–6]. Low protein intake reduces the digestibility of Ca [22, 23] and hence may influence the growth rate. Given some evidence on the effects of BCAA on homeostasis of Ca [16–18, 27] and positive effect of BCAA on growth of pigs fed with VLP diets [7, 12–14], it is unknown whether BCAA induced growth under protein restriction is linked with changes in Ca absorption/reabsorption. Here we examined whether supplementing VLP diets with BCAA more than recommended levels would influence the dynamics of Ca (re)absorption. Our study showed that relative to PC or NC: 1) supplementation of BCAA together with LAA at 25% or 50% more than recommended levels (i.e., LB25 and LB50) into VLP diet improved the Ca digestibility in last 3 weeks of study, 2) LB25 and LB50 reduced plasma VD_3_, but increased Ca and P concentration, 3) LB25 and LB50 decreased the transcript or protein abundance of CALB1 and CYP27B1 in gut, 4) LB50 tended to increase the protein abundance of VDR in jejunum, 5) LB50 decreased the transcript of *OCLN* and *PTH1R* in duodenum, 6) LB50 increased the transcript of *CYP24A1* and *PTH1R*, but decreased the transcript of *TRPV5* and LB25 increased the gene expression of *CYP24A1* and *SLC34A2* in kidney, 7) LB25 and LB50 reduced transcript of *CALB1* and *SLC17A4* in kidney. Overall, the supplementation of BCAA greater than recommended levels into the VLP diet improved Ca digestibility and increased plasma Ca and P concentration through regulating the transcellular and paracellular pathways of Ca absorption in the gut and Ca and P reabsorption in kidney.

It is well documented that not only protein intake but also dietary individual AA have modulatory impacts on Ca metabolism through the mechanisms related to Ca absorption, excretion, and hormonal changes [46, 47]. Whether BCAA supplementation in VLP diets changes the intestinal Ca digestibility and absorption is unclear. To our knowledge, this study is the first reporting the effect of supplementing BCAA and LAA on Ca digestibility, absorption, and reabsorption in young pigs fed with VLP diet. Supplementation of BCAA together with LAA more than recommended levels did not change the AFD of Ca and P in first two weeks of the study, but that improved the digestibility of these nutrients during the last three weeks of the experiment in pigs fed with VLP diets. The improved digestibility of Ca and P following BCAA supplementation over the course of study could be attributed to enhanced efficiency of gastrointestinal tract for nutrients absorption with age following weaning. The gastrointestinal tract of young pigs faces with interrupted nutrient digestibility in early-weaning stage which is mostly related to pH, enzyme secretion, and gut motility [48]. Although there were no differences among groups on digestibility of Ca and P in early stages of the study, BCAA supplementation improved the fecal consistency score and decreased the risk of post-weaning diarrhea in first week of the study. Unlike AFD of Ca and P, we failed to detect any differences among groups on AFD of N throughout the study. One caveat to our study is that nutrients digestibility were assessed by AFD method; however, future research should aim at estimating the standard ileal digestibility of Ca, P and N in pigs fed with protein deficient diets enriched with BCAA.

Supplementation of BCAA together with LAA higher than recommended levels (i.e., LB25 and LB50) increased plasma Ca and P concentrations. In support of our data, addition of BCAA keto acids into the VLP regimen of patients with chronic renal failure, significantly increased serum P levels [49]. The increased plasma Ca levels in pigs fed with VLP diets supplemented with BCAA could be due to greater digestibility of Ca in these groups. There is a paucity of data on specific effects of BCAA on nutrients absorption from gastrointestinal tract and their reabsorption in kidney. Supplementation of Ile and Leu, as well as Ala, Pro, and hydroxyproline has been shown to improve the Ca absorption in rats [27]. In broilers, Val deficiency resulted in leg abnormalities, low bone Ca levels and increased Ca excretion in urine [16, 17] and in rats fed with low protein diets, a mixture of essential AA including BCAA improved the bone strength and bone mineral density [18]. Other studies demonstrated that dietary protein could stimulate Ca absorption [50–52]. Plasma Ca concentration is tightly regulated not only by gastrointestinal tract and kidney, but also with bone under hormonal regulations [30, 53, 54]. Supplementation of BCAA along with LAA higher than recommended levels, tended to increase plasma ALP levels in this study. This is in line with previous study where infusion of an AA solution containing 45% BCAA to overstressed postoperative patients significantly increased ALP levels [55]. Increased plasma ALP could be suggestive of an elevated bone turnover that might be related with increased plasma Ca concentration in BCAA supplemented groups. Whether similar to dietary protein [56], BCAA supplementation increases the endogenous acid production and in an effort for buffering from bone as a reservoir of alkali an increased bone resorption occurs requires further research. These data provide evidence on the role of dietary BCAA in Ca homeostasis; however, none of previous studies have specifically investigated the effects of BCAA. Further research on the effect of all BCAA or individual BCAA on Ca absorption, and excretion and bone turnover will shed light on the role of these AA on nutrients homeostasis.

The mechanisms that underlie the increased Ca digestibility and intestinal absorption in response to BCAA supplementation during protein restriction is unknown. Amino acids are involved in Ca absorption through different mechanisms including alteration in the metabolism of enterocytes and activation of extracellular receptors or acceptors [47], activation of CaSR [57], which is expressed in the gut [58] and stimulating the secretion of gastric acid and gastrin [59, 60], which can solubilize the Ca in the gut and increase its absorption through improved bioavailability. Absorption processes for Ca across epithelial tissue occurs through paracellular and transcellular pathways [54]; however, relatively limited data are available on the effect of AA and BCAA on gene and protein expression of key molecules involved in paracellular and transcellular absorption of Ca in the gastrointestinal tract. BCAA-enhanced Ca transport in the gastrointestinal tract has been reported to be NHE3 pathway dependent [27]. NHE3 is an important apical membrane antiporter which is crucial for intestinal absorption of nutrients including Ca [61]. In our study, we only observed a numerical increase in protein abundance of NHE3 in jejunum. Here, for the first time we showed that pigs fed with protein-restricted diet supplemented with BCAA more than recommended levels (i.e., LB50) tended to increase VDR transcript and protein abundance in the gut. VDR, which is a member of nuclear receptor superfamily mediates the action of VD_3_ (1,25(OH)_2_D_3_) during transcriptional events in peripheral tissues, including intestine [62] and is important for normal Ca absorption [63]. The increased expression of VDR in gastrointestinal tract is suggestive of an increased vitamin D activity in the gut that might contribute to increased Ca digestibility and its increased concentration in plasma of pigs fed with VLP diets supplemented with BCAA. Given the higher abundance of VDR and increased digestibility and plasma concentration of Ca and a lower mRNA or protein abundance of PTH1R, CALB1, CYP27B1 and OCLN in LB50 pigs, it appears that a feedback regulatory mechanism is involved in this process. OCLN is involved in passive and paracellular Ca transport while PTH1R, CALB1 and CYP27B1 are associated with transcellular absorption of Ca [54]. A negative feedback mechanism has been described between increased plasma Ca concentration and reduced secretion of parathyroid hormone and VD_3_ that eventually lead to inhibiting the Ca absorption [64]. LB25 and LB50 reduced plasma VD_3_ in the current study. VD_3_ along with parathyroid hormone plays a crucial role in maintaining the Ca homeostasis through increasing the intestinal Ca absorption [53, 65]. The reduced plasma VD_3_ in LB25 and LB50 pigs is likely the result of a negative feedback loop that inhibits the release of this hormone along with parathyroid hormone and Ca absorption when blood Ca is elevated [64]. The mechanisms by which BCAA supplementation induces changes in Ca absorption are not completely understood and whether BCAA stimulate intestinal calcium transport through other potential pathways requires further investigation.

Calcium homeostasis is under coordinated regulation of intestinal, renal and bone physiology [66]. Little is understood on the mechanisms involved in Ca reabsorption when protein deficient diets are supplemented with BCAA. Aromatic AA, but not BCAA, have been reported to stimulate the Ca excretion through activation of CaSR in embryonic kidney cells [67]. In the current study, LB50 increased the transcript of *CYP24A1* and in kidney. The active form of vitamin D is converted to 24-hydroxylated inactive product under the action of 1,25-hydroxyvitamin-D_3_–24-hydroxylase encoded by *CYP24A1* gene [68]. The increased expression of CYP24A1 in kidney is suggestive of a decreased concentration of active form of vitamin D. As discussed earlier, LB50 decreased the plasma VD_3_ concentration as well. Reduced concentration and active form of VD_3_ might contribute to decreasing Ca absorption in gut and Ca reabsorption in kidney as a compensatory response to increased blood Ca concentration. PTH1R is involved in urinary excretion of phosphate in kidney [69]. The higher transcript of renal *PTH1R* in LB50 pigs is indicative of a greater excretion of phosphate in kidney in an effort to maintain the P homeostasis through reducing the increased plasma P levels. Further, LB50 decreased the transcript of renal *TRPV5*, *CALB1*, and *SLC17A4*, which are all involved in reabsorption of Ca and P [70, 71]. Decreased expression of TRPV5, CALB1 and SLC17A4 in kidney supports the notion that under a homeostatic regulation, Ca and P reabsorption is decreased to decline the plasma augmented Ca and P concentration in pigs fed with VLP diets supplemented with BCAA. To better understand the effect of supplemental BCAA on excretion of Ca and P under protein restriction, further research is warranted to assess the level of these nutrients in urine.

## Conclusions

To our knowledge, this is the first study assessing the effect of protein deficient diets supplemented with BCAA higher than recommended levels on Ca digestibility and pathways involved in its (re)absorption. Supplemental BCAA improved the digestibility of Ca and the concentration of Ca and P during protein restriction. Our data provide evidence that BCAA are acting via both transcellular and paracellular pathways in gastrointestinal tract and control renal Ca and P reabsorption to regulate the circulating Ca and P concentration. It is remained to be determined whether BCAA influence the Ca homeostasis through bone or other potential pathways in gastrointestinal tract.

## Data Availability

The datasets supporting the conclusions of this article are included within the article.

## Abbreviations

Ala: Alanine
ALP: Alkaline phosphatase
AA: Amino acids
AFD: Apparent fecal digestibility
BUN: Blood urea nitrogen
BCAA: Branched-chain amino acids
CALB1: Calbindin 1
Ca: Calcium
CaSR: Ca-sensing receptors
CLDN-1: Claudin-1
CLDN: Claudins
cDNA: Complementary DNA
CP: Crude protein
CYP24A1: Cytochrome P450 family 24 subfamily A member 1
CYP27B1: Cytochrome P450 family 27 subfamily B member 1
FGF-23: Fibroblast growth factor 23
Ile: Isoleucine
Leu: Leucine
LAA: Limiting amino acids
Lys: Lysine
Met: Methionine
min: Minute(s)
NHE3: Na^+^/H^+^ exchanger 3
N: Nitrogen
N1: Nursery phase 1
N2: Nursery phase 2
N3: Nursery phase 3
OCLN: Occludin
PTH: Parathyroid hormone
PTH1R: Parathyroid hormone 1 receptor
P: Phosphorus
PMCA1b: Plasma membrane Ca^2+^ ATPase 1b
Pro: Proline
qPCR: Quantitative PCR
SLC17A4: Solute carrier family 17 member 4
SLC34A1: Solute carrier family 34 member 1
SLC34A2: Solute carrier family 34 member 2
SLC34A3: Solute carrier family 34 member 3
Thr: Threonine
THRA: Thyroid hormone receptor alpha
TRPV: Transient receptor potential cation channel subfamily V
TRPV5: Transient receptor potential cation channel subfamily V member 5
TRPV6: Transient receptor potential cation channel subfamily V member 6
TBST: Tris-buffered saline containing 0.1% Tween-20
Trp: Tryptophan
Val: Valine
VLP: Very low-protein
VDR: Vitamin D receptor
VD_3_: Vitamin D_3_
ZO1: Zonula occludens 1
